# “Encephalopathy Only Stroke Codes” (EoSC) Rarely Result in Stroke as Final Diagnosis

**DOI:** 10.1155/2019/2105670

**Published:** 2019-02-11

**Authors:** Patrick M. Chen, Dawn M. Meyer, Robert Claycomb, Kunal Agrawal, Brett C. Meyer

**Affiliations:** ^1^Department of Neurosciences, Stroke Center, University of California San Diego, San Diego, CA, USA; ^2^Desert Regional Medical Center, Palm Springs, CA, USA

## Abstract

Stroke codes prompted by isolated encephalopathy often result in nonstroke final diagnoses but require intensive stroke center resources. We assessed the likelihood of “Encephalopathy only Stroke Codes (EoSC)” resulting in a true stroke (EoSC CVA+) final diagnosis. 3860 patients were analyzed in a prospective stroke code registry from 2004 to 2016. EoSC was defined using a standard and an exploratory definition. Definition 1 included EoSC patients as stroke codes where NIHSS was nonzero for LOC questions (questions la, 1b, and lc) but remainder of the NIHSS was zero. Definition 2 included the same definition but allowed symmetric pairings on motor questions (5a/5b, 6a/6b, or Question 4 scoring a 3). Groups were assessed for final diagnosis of stoke (EoSC CVA+) or not stroke (EoSC CVA-). EoSC accounted for 60/3860 (1.55%) of total stroke codes. EoSC CVA+ was found in 5/3860 (0.13%) of all stroke codes, 5/60 (8.33%) of EoSC stroke codes, and 5/1514 (0.33%) of all strokes. For Definition 2, EoSC accounted for 96/3860 (2.5%) of total stroke codes. EoSC CVA+ was found in 9/3860 (0.23%) of all stroke codes, 9/96 (9.38%) of EoSC stroke codes, and 9/1514 (0.59%) of all strokes. On multivariable logistic regression analysis, diabetes was the highest predictor of stroke (p=0.05). Encephalopathy only Stroke Codes only rarely result in cases with a true final diagnosis of stroke (EoSC CVA+), accounting for 0.1-0.2% of all stroke codes and 8-9% of EoSC stroke codes. This may have important significance for mobilization of limited acute stroke code resources in the future.

## 1. Introduction

Acute stroke code protocols are widely used and have improved response times and rt-PA administration in acute stroke. Stroke code systems' effectiveness hinges on rapid application and mobilization of limited resources for every stroke code. Previous studies suggest that stroke mimics account for 30% of overall stroke codes [1]. Presence of acute encephalopathy may account for a substantial portion of all stroke codes, either in isolation or in association with other neurologic findings [2]. While stroke is classically characterized by a focal and unilateral deficit, some stroke types (bilateral, basilar, or thalamic) can present with encephalopathy or even bilateral deficits. Previous studies have suggested that altered mental status is a poor indicator of ischemic stroke diagnosis [2–6]. There is a gap in the literature related to whether isolated encephalopathy has the same low likelihood of predicting final stroke diagnosis. This analysis assessed the likelihood that “Encephalopathy only Stroke Codes” without other focal neurologic deficit (EoSC) would result in a final diagnosis of true stroke (EoSC CVA+).

## 2. Methods

We retrospectively assessed consecutive patients in a prospectively collected, IRB approved, stroke code registry, from June 2004 to June 2016. We analyzed baseline characteristics of age, sex, race, initial NIH Stroke Scale (NIHSS), diabetes, hypertension, coronary artery disease (CAD), atrial fibrillation (AFib), blood pressure, alcohol, smoking, and relevant stroke code evaluation window time points for all patients and compared them between specific groups (“Encephalopathy Only” (EoSC) vs. “non-Encephalopathy Only” (non-EoSC)).

**Figure 1:**
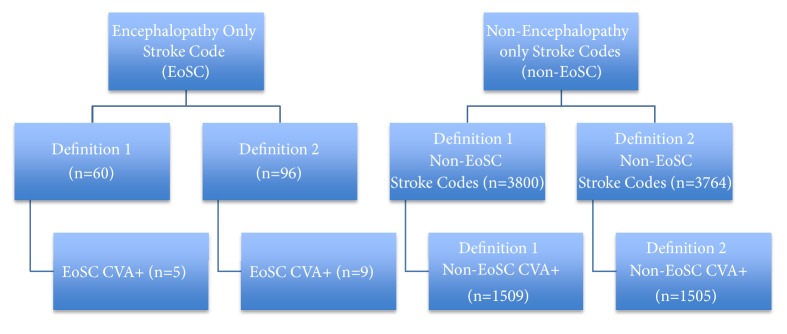
*Study Definitions*. Schematic showing study definitions. Stroke codes are first defined as encephalopathy only (EoSC) or other stroke codes (non-EoSC). EoSC were put into two different definitions based on NIHSS: one with only mental status changes (Definition 1) and another allowing for symmetric motor findings (Definition 2). Under each definition there is a final diagnosis of stroke (EoSC CVA+) which is compared to non-EoSC stroke positive diagnoses (non-EoSC CVA+).

Standard Definition 1 (Figure 1) included any stroke code patient where the NIHSS showed evidence of encephalopathy by scoring positive on any of the 3 levels of consciousness (LOC) questions (Question la= LOC, Question 1b= LOC-Questions, or Question 1c= LOC-Commands) while the remainder of the NIHSS items were scored without deficit (score=0). The exploratory Definition 2 was also included to account for an “encephalopathy effect” on NIHSS scoring. This accounted for a potential >0 score simply due to lack of following commands. This definition included a patient where the motor NIHSS scores were >0 but scored as no more than 1 point difference for Question 5a vs. 5b (motor left arm vs. motor right arm), or for Question 6a vs. 6b (motor left leg vs. motor right leg), or a score of 3 on Question 4 (complete or bilateral facial paralysis) (Figure 1). This included pairings of 1/1, 2/2, 3/3, 4/4, 4/3, 3/4, 2/3, 3/2, 1/2, 2/1, and 0/1. This expanded definition is based on the low likelihood of strokes having bilateral findings and the low likelihood of posterior circulations strokes not also having other scorable signs if the motor exam was truly affected bilaterally (unilateral facial droop, ataxia, or gaze preference). We then compared groups for final diagnosis of stroke (CVA+) using final database diagnosis (based on discharge ICD coding).

Data was examined for frequencies and distribution. Baseline demographics were compared via chi-squared (nominal), Fischer's exact (nominal), t-test (continuous), or Mann–Whitney U (ordinal) as appropriate to the data. Correlation with Spearman (nominal) or Pearson's (continuous) was utilized to assess relationships between EoSC (yes/no) and the variables assessed in the correlation. All variables with a p <0.1 were included in a logistic regression to assess variables significantly associated with the diagnosis of stroke or no stroke. A p value of <0.05 was considered significant.

## 3. Results

For the primary analysis, a total of 3,860 stroke codes were identified. Baseline demographics for all stroke codes are presented in Table 1(a). In the EoSC stroke code group, there was a higher percentage of female (40/60 (66.67%) vs. 1801/3800 (47.39%); p=0.004) and older age patients (70.65 vs. 66.3; p=0.04). There were no differences in acute stroke time window metrics. As expected by the EoSC definitions, total NIHSS was lower in the EoSC group vs. the non-EoSC group (2 vs. 9; p<0.001). EoSC accounted for 60/3860 (1.55%) of all stroke codes. Table 1(b) shows the analysis for patients with final true diagnosis of stroke only (non-EoSC CVA+ vs. EoSC CVA-). When assessing those patients with final diagnosis of stroke, the total NIHSS was also lower in the EoSC CVA+ group vs. the non-EoSC CVA+ group (2 vs. 11; p=0.004). EoSC CVA+ accounted for 0.13% of all stroke codes, 8.33% of EoSC stroke codes, and 0.33% of all strokes. For the adjusted analysis, baseline NIHSS, age, and gender were included as covariates since they were unbalanced between groups at 0.1 significance level. No variables were found to be associated with final diagnosis of stroke; therefore no multivariate model was fit.

For the exploratory Definition 2 analysis, baseline characteristics of all stroke codes under this definition are noted in Table 2(a). In the EoSC stroke code group, there was a higher percentage of female (60.42% vs. 47.37%; p=0.01) and older age patients (71.3 vs. 66.2; p=0.004). There were no differences in acute stroke time window metrics. The total NIHSS was lower (4 vs. 9; p<0.001) in the EoSC stroke code group vs. the non-EoSC group overall. EoSC accounted for 96/3860 (2.5%) of all stroke codes. Table 2(b) shows the demographics for patients with final true diagnosis of stroke only (non-EoSC CVA+ vs. EoSC CVA-) under Definition 2 analysis. There was a higher percentage of diabetes in the EoSC CVA+ group than in the non-EoSC CVA+ group (5/9 (55.56%) vs. 369/1505 (24.52%); p=0.05). There were no differences in acute stroke time window metrics. As expected by the EoSC definitions, total NIHSS was also lower (4 vs. 12; p=0.01) in the EoSC CVA+ group vs. the non-EoSC CVA+ group. EoSC CVA+ accounted for 9/3860 (0.23%) of all stroke codes, 9/96 (9.38%) of EoSC stroke codes, and 9/1514 (0.59%) of all strokes. For the adjusted analysis, as noted in Table 3, diabetes and ethnicity were found to be associated with final diagnosis of stroke, though on multivariable logistic regression analysis, only diabetes maintained significance (p=0.05).

## 4. Discussion

Altered mental status has been described as a poor predictor of ischemic stroke but still a high risk among the neurologic disease population [5, 6]. Acute stroke codes are often found to have isolated encephalopathy but there is limited literature on the frequency with which this results in stroke code activation, frequency of final stroke diagnosis, or how this stroke code activation affects limited acute stroke code resource availability. In our study, EoSC occurred in a reasonable percentage of stroke codes for Definition 1 (1.6%) and even higher percentage for the more exploratory Definition 2 (2.5%). Though other studies have shown a higher rate of 40% [3–6], this is likely due to our restricted definition of “encephalopathy only”. In our study, final stroke diagnosis in EoSC (EoSC CVA+) was noted in a small percentage of EoSC stroke codes (8-9%) but a very small percentage of overall stroke codes (0.13%), highlighting the fact that patients where the only finding is isolated encephalopathy do not often result in a final diagnosis of stroke.

It is not unexpected that EoSC stroke codes had a higher percentage of older patients as encephalopathy is more common in the elderly [7]. These differences were not found in true stroke (EoSC CVA+) patients. We found diabetes to be the only significant factor to predict stroke within the EoSC CVA+ cohort (using Definition 2) but did not find the expected correlation with AFib or cardiac history [8]. These findings may be due to the resultant small eventual sample size of EoSC diagnosed CVA+ patients.

Though stroke usually presents as a focal neurologic deficit, the fear of an unusual presentation of stroke may drive practitioners to call a stroke code in even these cases where stroke is unlikely. Though our analysis showed that final EoSC CVA+ diagnosis was rare (0.1-0.2% of all codes), this should not result in limiting acute stroke code resources for patients with encephalopathy. Practitioners may use this information to gauge when to activate a stroke code, or stroke specialists may use this information to consider how to allocate limited triage resources when multiple codes occur.

Another clinical concern is the fear of missing posterior circulation strokes which make up the majority of missed strokes and can result in a poor prognosis [9]. For this concern, it should be noted that missed diagnoses of stroke in encephalopathy cases can be correlated with having missed subtle localizing signs on exam [6] and that lethargy and confusion account for only the minority of posterior strokes [9, 10]. An NIHSS based classification may not accurately estimate the occurrence of true isolated encephalopathy, especially if aphasia or neglect are scored as confusion or vice versa. This limitation is dependent on accurate documentation and excellence of neurologic exam. A strict NIHSS based definition of encephalopathy provides a proxy evaluation of encephalopathy code incidence. A NIHSS based classification like that used in this study may also be a plausible for triaging stroke code activation. This will require prospective validation and also assumes clinicians are comfortable teasing out exam nuances [9]. Finally, posterior circulation strokes with encephalopathy could have true bilateral findings on arm or leg weakness (arguing against our exploratory definition) but these posterior circulation strokes would be expected to have cranial nerve abnormalities or ataxia, which would have been scored on the NIHSS and thus excluded from our population.

Other limitations of our analysis include the relatively small sample size, our retrospective design, and our reliance on the database entry for NIHSS scoring and final diagnosis reporting. The key limitation to generalizing the use of these limited results would be the “lost chance standard” in rt-PA [11]. Missing even a single stroke, by not activating a stroke code, would result in a patient potentially being denied the possibility of a life- saving therapy. As such, the results should be interpreted without overgeneralization.

Considering these results, the low likelihood that EoSC codes result in final stroke diagnosis may have important significance for mobilization of limited acute stroke code resources. Subsequent planned analyses will include manual chart review of all codes to determine the frequency of EoSC overall, the percentage of those who received therapies, what the reason for the code was, and the resultant stroke subtype.

## Figures and Tables

**(a):** 

	Non-EoSC (n= 3800)	EoSC + (n=60)
*Age (SD)∗*	66 (16)	70 (16)
*Male (n, *%)*∗*	1999 (52)	20 (33)
*White Race (n, *%)	3018 (79)	52 (86)
*DM (n, *%)	903 (23)	11 (18)
*Atrial fibrillation (n, *%)	700 (18)	12 (20)
*Baseline NIH Stroke Scale (SD)∗*	9 (10)	2 (1)
*Baseline Systolic Blood Pressure (SD)*	150 (30)	151 (26)

**(b):** 

	Non-EoSC CVA + (n=1509)	EoSC CVA+ (n=5)
*Age (SD)*	70 (15)	72 (13)
*Male (n, *%)	849 (56)	2 (40)
*White Race (n, *%)	1236 (81)	4 (80)
*DM (n, *%)	372 (24)	2 (40)
*Atrial fibrillation (n, *%)	396 (26)	2 (40)
*Baseline NIH Stroke Scale (SD)∗*	11 (10)	2 (1)
*Baseline Systolic Blood Pressure (SD)*	154 (30)	159 (12)

Demographics of (a) all non-encephalopathy stroke codes (non-EoSC) vs. encephalopathy only codes (EoSC) and (b) all non-encephalopathy stroke codes that were stroke positive (non-EoSC CVA +) vs. EoSC stroke positive (EoSC CVA+) using Definition 1 (positive findings in 1a, 1b, or 1c only). *∗* indicates statistical significance (p<.05).

**(a):** 

	Non-EoSC (n=3764)	EoSC + ( n = 96)
*Age (SD)∗*	66 (16)	71 (14)
*Male (n, *%)*∗*	1981 (52)	38 (40)
*White Race (n, *%)	2992 (80)	78 (81)
*DM (n, *%)	892 (23)	22 (23)
*Atrial fibrillation (n, *%)	695 (18)	17 (18)
*Baseline NIH Stroke Scale (SD)∗*	9 (10)	4 (4)
*Baseline Systolic Blood Pressure (SD)*	149 (30)	145 (26)

**(b):** 

	Non=EoSC CVA + (n=1505)	EoSC CVA + (n=9)
*Age (SD)*	70 (16)	71 (14)
*Male (n, *%)	848 (56)	3 (33)
*White Race (n, *%)	1233 (82)	7 (77)
*DM (n, *%) *∗*	369 (25)	5 (56)
*Atrial fibrillation (n, *%)	396 (26)	2 (22)
*Baseline NIH Stroke Scale (SD)∗*	12 (10)	4 (3)
*Baseline Systolic Blood Pressure (SD)*	154 (30)	142 (14)

Demographics of (a) all non-encephalopathy stroke codes (non-EoSC) vs. encephalopathy only codes (EoSC) and (b) all non-encephalopathy stroke codes that were stroke positive (non-EoSC CVA +) vs. EoSC stroke positive (EoSC CVA+) using Definition 2 (Definition 1 but also allowing symmetric 5a/5b, 6a/6b, or question 4 scoring a 3). *∗* indicates statistical significance (p<.05).

**Table 3:** Predictors of stroke in EoSC patients.

	Odds Ratio	p-value
Diabetes	5.8	0.04
Hispanic Race	2.6	0.32

Definition 2 under multivariate analysis. For Definition 1 and 2, age and cardiovascular factors (HTN, atrial fibrillation coronary artery disease) did not show statistical significance.

## Data Availability

The data used to support the findings of this study are available from the corresponding author upon request.
